# Unconscious Processing of Negative Animals and Objects: Role of the Amygdala Revealed by fMRI

**DOI:** 10.3389/fnhum.2016.00146

**Published:** 2016-04-05

**Authors:** Zhiyong Fang, Han Li, Gang Chen, JiongJiong Yang

**Affiliations:** ^1^Department of Psychology and Beijing Key Laboratory of Behavior and Mental Health, Peking UniversityBeijing, China; ^2^Scientific and Statistical Computing Core, National Institute of Mental Health (NIMH)/National Institutes of Health (NIH)/Department of Health and Human Services (DHHS)Bethesda, MD, USA

**Keywords:** category, amygdala, awareness, emotion, context

## Abstract

Previous studies have shown that emotional stimuli can be processed through the amygdala without conscious awareness. The amygdala is also involved in processing animate and social information. However, it is unclear whether different categories of pictures (e.g., animals, objects) elicit different activity in the amygdale and other regions without conscious awareness. The objective of this study was to explore whether the factors of category, emotion and picture context modulate brain activation for unconscious processing. Pictures denoting different nonhuman animals and objects in negative and neutral emotional valences were presented using a sandwich-masking paradigm. Half of them were presented with human-related information in the contexts, and half were not. Our results showed significant interaction among category, emotion and context in the amygdala and subcortical regions. Specifically, negative animals elicited stronger activation in these regions than negative objects, especially with human contexts. In addition, there were different correlation patterns between the amygdala and cortical regions according to whether they included human context. There were limited activations in cortical category-related networks. These results suggest that the amygdala and subcortical regions dominantly process negative animals, and contextual information modulates their activities, making threatening stimuli that are most relevant to human survival preferentially processed without conscious awareness.

## Introduction

Many studies have shown that the amygdala plays an important role in emotional processing under both conscious and unconscious conditions (for reviews, see Pessoa and Adolphs, 2010; Tamietto and de Gelder, 2010). When stimuli are presented without conscious awareness, the amygdala shows enhanced activation for negative relative to neutral stimuli in healthy participants (e.g., Morris et al., 1998; Whalen et al., 1998; Jiang and He, 2006; Williams et al., 2006). Patients with amygdala lesions demonstrate comparable physiological responses for masked emotional pictures (30 ms) in comparison to neutral pictures (e.g., Glascher and Adolphs, 2003). In addition, the amygdala activation is correlated with that in subcortical regions, including the pulvinar and anterior nucleus of the thalamus (Morris et al., 1999; Williams et al., 2006). These results suggest that unconscious response to emotional, especially negative, stimuli depends on the amygdala and subcortical regions (Tamietto and de Gelder, 2010).

In addition to being involved in emotional processing, the amygdala is also more responsive to animate relative to inanimate stimuli. The preparedness model (Seligman, 1970; Ohman and Mineka, 2001) emphasizes the role of the stimulus category in emotional processing. It is proposed that fear is more readily learned and resistant to extinction for threatening stimuli that are related to our evolutionary ancestors (e.g., the phylogenetic fear of snakes or spiders) than for those that have only recently emerged in our cultural history (e.g., the ontogenetic fear of guns or motorcycles; for reviews, see Ohman and Mineka, 2001; Mineka and Ohman, 2002). Studies have found that the amygdala responds more strongly to animate stimuli than inanimate stimuli in neural recording of patients (e.g., Mormann et al., 2011; Rutishauser et al., 2011) and in fMRI studies (e.g., Yang et al., 2012a; Coker-Appiah et al., 2013).

However, it remains unclear whether different categories of negative stimuli modulate activation in the amygdala and other brain regions for unconscious processing. Animate categories of stimuli may be more likely to induce fear. For example, negative animal pictures showed stronger skin conductance responses (SCR) than objects under the unconscious condition (Tan et al., 2013). The preparedness theory hypothesized that, compared to the ontogenetic category (e.g., guns), the phylogenetic category (e.g., snakes) could be processed without conscious awareness and this processing would be amygdala dependent (Ohman and Mineka, 2001; Mineka and Ohman, 2002). So far, few neuroimaging studies have directly manipulated stimulus category during unconscious emotional processing. For example, when masked pictures of snakes and spiders were compared with those of mushrooms (Carlsson et al., 2004), the amygdala was strongly activated. However, it is noteworthy that snakes and mushrooms differ in both valence and category, making it difficult to interpret the results as emotional or category effect.

The amygdala is also activated by stimuli depicting social information (Adolphs, 2010; Frith and Frith, 2012) when stimulus information appear in a background involving human-related information (Norris et al., 2004), or when stimuli can be interpreted as social interaction or human-related (Wheatley et al., 2007; Sakaki et al., 2012). Category and social contexts interact in emotional processing, because negative animal and object pictures elicit comparable activation in the amygdala when a human or human body is included as contextual information (Cao et al., 2014). There is some evidence suggesting that social cues could be processed unconsciously (Adolphs, 2010; Frith and Frith, 2012). For example, during unconscious processing, faces turned towards the viewer broke through suppression faster than faces that were turned away (Gobbini et al., 2013), whereas nonsocial cues interfered with the target detection and led to higher subjective threshold (Ruderman and Lamy, 2012). Crouzet et al. (2012) found that contextual information tended to bias responses to animate objects (but not vehicular responses) as early as 160 ms after stimulus onset. Despite this, opposite evidence existed as well (e.g., Tan et al., 2013), which showed that animal pictures elicited stronger SCR than objects under the unconscious condition, irrespective of contextual information. Therefore, the extent to which social contexts modulate amygdala activation during unconscious processing is unclear.

The current study addressed these questions by presenting subjects with pictures of animals and objects in negative and neutral valences during functional magnetic resonance imaging (fMRI). To ensure that subjects processed the pictures without conscious awareness, a sandwich-masking paradigm was adopted and *post hoc* awareness detection was performed. To dissociate the effects of emotion and category, valence and arousal levels were matched in animal and object categories. To examine the effect of context on brain activity, picture contexts including human or nonhuman information were manipulated. Main effects of the emotion, category and context and their interactions were analyzed. In addition to the voxel-wise approach, we also applied a connectivity analysis in order to clarify the patterns of inter-area functional correlation in different experimental conditions (e.g., Jiang and He, 2006; Williams et al., 2006). The correlation analysis could further identify whether animals and objects elicited different brain connectivity during unconscious processing of emotionally laden animals and objects. We hypothesized that negative pictures would elicit stronger amygdala responses than neutral pictures. In addition, based on the preparedness theory (Ohman and Mineka, 2001) and previous behavioral and psychophysiological findings (Ohman and Soares, 1993; Tan et al., 2013), we hypothesized that animal images would lead to stronger amygdala responses than pictures of objects. Finally, we predicted that the subcortical and cortical regions would show differential activity for animal relative to object pictures, especially for negative pictures (Isbell, 2006; Tamietto and de Gelder, 2010).

## Materials and Methods

### Subjects

Sixty-two healthy, right-handed subjects (28 males) participated in the study, with a mean age of 22.54 years (standard deviation (SD): 2.75). Of these subjects, 21 participated in emotional rating (10 males), 18 in familiarity rating (7 males), and the other 23 in the fMRI experiment (11 males). Among the 23 subjects who participated in the fMRI experiment, four were excluded from fMRI analysis due to large head motion (two subjects) and high awareness level (two subjects, see details in the “Results” Section). All subjects were native Chinese speakers, and gave written informed consent in accordance with the procedures and protocols approved by the Institutional Review Board of the Department of Psychology, Peking University.

### Stimuli

**Figure 1:**
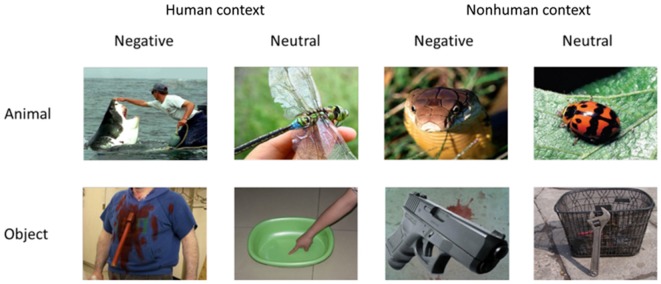
**Stimulus example of the experiment**.

Three within-subject factors were considered in the study with a 2 × 2 × 2 structure: context (with [H] or without human context [NH]), emotion (negative, neutral) and category (nonhuman animals, objects; Figure 1). The combinations of the three factors made up eight experimental conditions (i.e., animals and objects in H-negative, H-neutral, NH-negative, NH-neutral conditions). The stimuli in the fMRI experiment consisted of 240 colorful, nameable experimental pictures (30 per condition) with a resolution of 640 × 480 pixels. All target images in the stimuli (i.e., the item defining the category of the stimulus, e.g., a snake) were depicted with or without human context (or human parts, e.g., an object held in a hand). In the pictures, human contexts interacted with the animal/object. In some negative pictures, the animal or the object had the action/potential to harm a human (e.g., a bear running at a human). In other negative pictures, human context handled the animal or the object (e.g., a snake is put on the hand). Low-level visual features (e.g., luminance, contrast, saturation of each color channel), picture size, position of focal object and context (i.e., central/peripheral, left/right) and contextual information (e.g., human face, hands) were also matched across categories (Yang et al., 2012b). Control stimuli were scrambled pictures, which were consisted of phase-scrambled images of each picture that preserved the color and the spatial frequency of the original image.

Analyses of the rating data by 21 subjects confirmed that categories of animals and objects did not significantly differ on valence (1 = very unpleasant to 9 = very pleasant) and arousal (1 = very calming to 9 = very arousing; Table 1). By design, negative pictures were more negative and more arousing than neutral pictures, *F*_(1,20)_ = 230.73, *p* < 0.001, and *F*_(1,20)_ = 99.26, *p* < 0.001. Pictures with human contexts were more negative and more arousing than those without, *F*_(1,20)_ = 58.27, *p* < 0.001, and *F*_(1,20)_ = 78.62, *p* < 0.001. Rating of animal and object pictures did not significantly differ for valence, *F*_(1,20)_ = 0.09, *p* = 0.76, nor for arousal, *F*_(1,20)_ = 3.44, *p* = 0.08. The interactions related to category were not significant, *p*s > 0.05 (i.e., interaction between category and emotion, category and context, and three-way interaction). Particularly, there was no significant difference between animals and objects in each condition comparison (i.e., H-negative, H-neutral, NH-negative, NH-neutral) for both valence (*p*’s > 0.30) and arousal (*p*’s > 0.10) ratings. Thus, affective levels would not confound the activation related to category.

**Table 1:** **Rating results**.

		Human context				Nonhuman context			
		Negative		Neutral		Negative		Neutral	
		Animal	Object	Animal	Object	Animal	Object	Animal	Object
Valence	Mean	3.08	2.95	4.84	4.75	3.65	3.82	5.10	4.94
	SD	0.79	0.53	1.02	0.41	1.01	0.79	1.19	0.37
Arousal	Mean	6.61	6.43	4.51	4.22	5.89	5.56	4.02	3.82
	SD	1.20	1.35	1.24	1.39	1.19	1.18	1.31	1.48
Familiarity	Mean	3.43	3.71	4.15	4.39	3.59	3.72	4.29	4.55
	SD	1.41	1.33	1.51	0.74	1.39	1.29	1.54	0.75

The familiarity was also matched between categories. Subjects evaluated how often they saw or thought of the focal object (i.e., an animal or object) in their daily life (1 = least familiar; 7 = most familiar). The results showed that category effect was not significant, *F*_(1,17)_ = 1.66, *p* = 0.22. Neutral pictures were more familiar than negative pictures, *F*_(1,17)_ = 17.65, *p* = 0.001, and pictures without human contexts were more familiar than those with human contexts, *F*_(1,17)_ = 12.09, *p* = 0.003. There were no significant category-related effects, *p*’s > 0.20, and the familiarity rating scores were comparable between animals and objects in each condition, *p*’s > 0.10. Thus, familiarity rating was not a confounding factor that might influence the category effect in the fMRI analysis.

### fMRI Procedure

**Figure 2:**
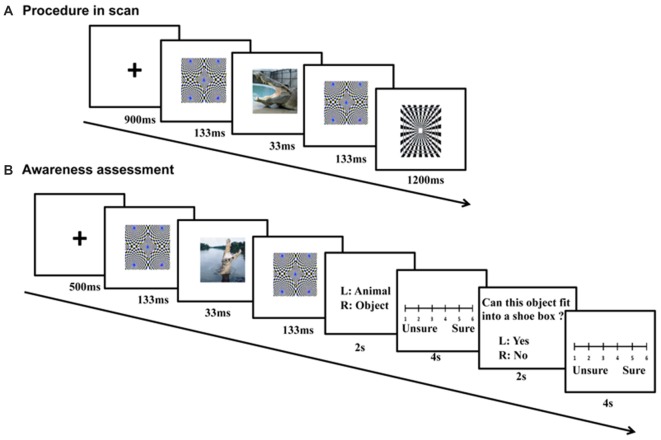
**Procedure of fMRI scanning and follow-up awareness assessment.** For each fMRI trial **(A)**, a cross (900 ms), a forward mask (133 ms), a stimulus picture (33 ms), and a backward mask (133 ms) were presented sequentially. During the awareness assessment **(B)**, the sandwich-masked picture was presented and subjects were asked to judge whether the content of the masked picture was a nonhuman animal or an object, and whether the concept depicted by the picture could be placed in a shoe box based on its actual size.

Pictures were clustered into blocks by context, emotion and category, with each of the 2 × 2 × 2 conditions having two blocks. In each block, there were 20 images (15 different stimulus pictures and five squares). For each trial, a cross “+” (900 ms), a forward mask (133 ms), a stimulus picture (33 ms, refresh rate of the Samsung monitor was set at 60 Hz), and a backward mask (133 ms) were presented sequentially (Figure 2A). An identical masking picture served as the forward and backward mask in each trial. Following the backward mask, another cross “+” appeared. A black-and-white square was randomly presented in five trials within a block after the backward mask, and its time was not counted into the trial timeline (Figure 2). The participants were asked to press a button within 1200 ms when they saw the square. In other words, they had to detect the black-and-white square if they saw it after the masks. Each trial lasted for 1200 ms, and each block for 24 s. The 16 picture blocks and 16 scrambled blocks were pseudo-randomly assigned to four runs, with the picture conditions and backgrounds balanced across runs. In addition, the picture and scrambled blocks were interleaved in each run. Because four additional TRs (two before the first block and two after the last block) were inserted for each run, each run lasted for 240 s and the entire experiment lasted for about 16 min. The orders of the blocks and runs were counterbalanced across subjects. Subjects were also asked to fill in state and trait questionnaire of the State Trait Anxiety Inventory (STAI), once before and once after the scanning.

### *Post hoc* Awareness Assessment

The awareness levels of the participants were tested within 1 week after the fMRI scanning using semantic judgment tasks (Figure 2B). The same stimuli and sandwich-masking manipulations were employed as those in the fMRI procedure, but the stimuli were presented in different pseudo-random orders (for blocks and runs) and a Iiyama monitor was used (refresh rate = 60 Hz). Participants were required to make two choices after each masked presentation: whether the content of the masked picture was a nonhuman animal or object, and whether the concept depicted by the picture could be placed in a shoe box based on its actual size. Each choice was followed by a confidence rating associated with the response on a 6-point scale.

### MRI Acquisition

MRI data were collected on a Siemens Trio 3T scanner (Magnetom Trio) with functional and then anatomical data acquired respectively using a gradient echo, echo-planar imaging (EPI) and a high-resolution MP-RAGE sequence (repetition time (TR) = 7.6 ms, flip angle = 6°, field of view (FOV) = 22 cm, matrix = 256 × 256, resolution = 1 × 1 × 1.2 mm^3^). The parameters for the EPI sequence were TR = 3 s, echo time (TE) = 40 ms, flip angle = 90°, gap = 1 mm, FOV = 24 cm, matrix = 96 × 96, slice = 34 and resolution = 2.5 × 2.5 × 4 mm^3^.

###  fMRI Data Analysis

AFNI was used for pre-processing imaging data and statistical analysis (http://afni.nimh.nih.gov. Cox, 1996). The EPI volumes were registered, smoothed with an RMS width of 3 mm, and scaled to a voxel-wise mean of 100. Multiple regression analysis was used to estimate the response to each condition compared with the scrambled baseline. The model included eight regressors of interest, each of which was created by convolving a gamma variate with the stimulus timing under each condition, six regressors of no interest (motion parameters), and 2nd order polynomials (slow drift). Anatomical images and the volumes of effect estimates from the regression analysis were then warped into the standard stereotaxic space of the Talairach and Tournoux (1988) atlas.

For group analysis, a voxel-wise analysis of variance (ANOVA) was performed with context, emotion and category as three within-subjects factors (voxel-wise *p* < 0.02, two-tailed). The reported statistics were all corrected by multiple comparisons at a cluster-level *P*-value of 0.05 in cortical regions (volume = 544 mm^3^) and the amygdala (small volume correction, volume = 131 mm^3^). The amygdala of each subject was manually drawn and averaged as the anatomical mask to confine the activation located within the amygdala. To further identify whether animals and objects elicited different brain connectivity during unconscious processing, the psychophysiological interaction analyses (PPI, Friston et al., 1997; Gitelman et al., 2003) were performed in which the two seed regions were created as a 5 mm-diameter sphere centered on the peak voxel within the left and right amygdala, and the average time series from each seed was extracted from the dataset with baseline, slow drift and head motion removed. The PPI inference at the group level was made through a one-sample *t*-test (*p* < 0.05, corrected, two-tailed).

## Results

### Behavioral Results

During scanning, subjects were highly accurate when performing the detection task. However, accuracy and reaction time did not show any significant effect in category, emotion, or their interaction (all *F*’s < 1.0, *p*’s > 0.20). Subjects had comparable STAI state before (32.1 ± 7.5) and after (32.3 ± 8.4) the scanning (*p* > 0.80), and their trait anxiety scores were within the normal range (37.4 ± 6.5).

For the awareness assessment, the receiver operating characteristic (ROC) scores were converted from the participants’ responses and confidence ratings, and the areas under the ROC curves (A′) were computed for each participant and subsequently entered into SPSS for analysis, in which the subjects were considered as being unaware when their detection task performance was at the chance level (i.e., the A′ values were not significantly different from the chance level of 0.5) in category and size judgments.

The results showed that in the category judgment task, the A′ values for two subjects were higher than the chance level (area = 0.57 and 0.59, *p*’s < 0.05), so their data were excluded from further analysis. The A′ scores for all remaining subjects were not significantly larger than the chance level (0.5; area = 0.48 ± 0.06; *t*_(18)_ = −2.11; *p* < 0.05). Subjects also performed at chance level in judging whether the picture was larger than a shoebox (area = 0.50 ± 0.04; *t*_(18)_ = 0.93, *p* = 0.10). In addition, we analyzed the A′ for H-negative, H-neutral, NH-negative, NH-neutral conditions, and the results confirmed that the A′s were not significantly different from the chance level in each condition (*p*’s > 0.10). We also performed the ANOVAs with emotion and context as factors for the A’ analysis. The results showed that for both semantic tasks, the main effects of emotion and context, and their interactions were not significant (*F*’s > 2, *p*’s > 0.10). These results confirmed that the subjects processed the semantic features of pictures without conscious awareness for different conditions.

### Main Effects of Emotion, Category and Context

We reported the fMRI results for main effects and their interactions. The interactions between emotion and context, category and context were not significant in the amygdala (*p*’s > 0.05; see Supplementary Tables 1, 2), so only the interaction of category and emotion, and three-way interactions were reported in the text.

**Figure 3:**
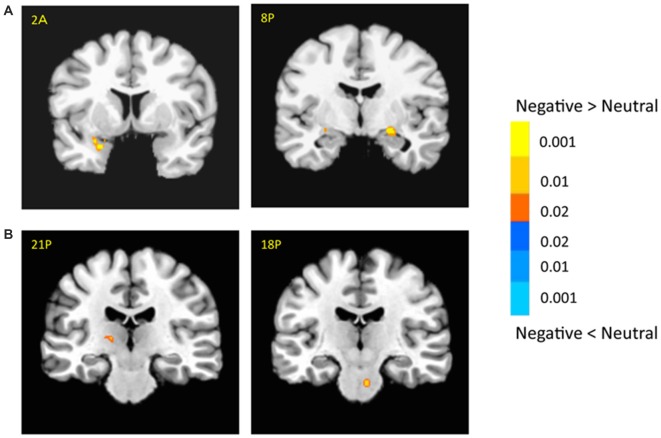
**Effects of emotion in the amygdala (A) and subcortical regions (B).** The color bar represents the contrast of negative vs. neutral (with warm color as negative > neutral), and represents *p*-values for *t*-statistics. The left is on the left side. The number/alphabet combinations shown at the top left corner of each brain image refer to the *y*-coordinates in the standard Talaraich atlas (e.g., Figure 2A refers to 2 mm anterior to the AC-PC line).

The bilateral amygdala showed stronger activation for negative pictures than for neutral pictures (i.e., emotional effect; left: −22, −1, −9; *t*_(18)_ = 6.54; *p* < 0.001; right: 21, −9, −9; *t*_(18)_ = 3.64; *p* < 0.005; Figure 3A), which was consistent with previous findings (for review, see Tsuchiya and Adolphs, 2007). In addition, there were stronger activations in the subcortical and cortical regions for negative vs. neutral pictures, including the right caudate (14, 16, 9; *t*_(18)_ = 4.03; *p* < 0.001), left thalamus (−14, −9, 1; *t*_(18)_ = 3.53; *p* < 0.005) and superior collicus (SC; 11, −24, −9; *t*_(18)_ = 2.88; *p* < 0.01; Figure 3B, Supplementary Table 1). The cortical activation for emotional effect was located in the posterior cingulate cortex (PCC), postcentral gyrus and precuneus. Although the right superior temporal sulcus (STS, 39, −54, 9; *t*_(18)_ = 3.72; *p* < 0.005) showed stronger emotional effect, the cluster size was relatively small and failed to survive after the rigorous multiple correction.

**Figure 4:**
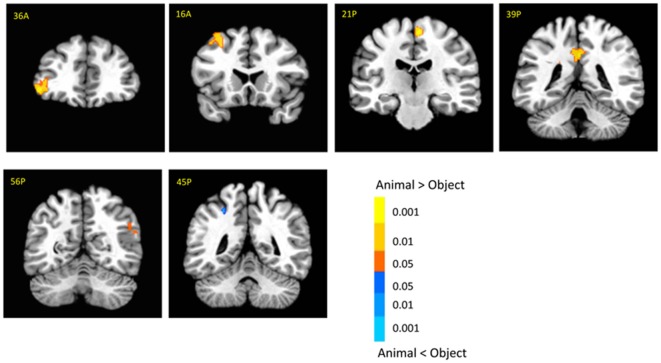
**Cortical activation for category effect.** Animal pictures elicited stronger activation than objects in the left anterior prefrontal cortex (PFC), left superior PFC, mPFC, anterior cingulate cortex (ACC), angular gyrus and temporal gyrus, and objects elicited stronger activation than animals in the left intraparietal lobe (IPL). The color bar represents the contrast of animal vs. object (with warm color as animal > object), and represents *p*-values for *t*-statistics. The left is on the left side. The number/alphabet combinations at the top left corner of each brain image refer to the *y*-coordinates in the standard Talaraich atlas.

There was no significant activation in the amygdala for the main effect of category, but there were stronger activations for animals than objects in the brainstem (6, −14, −29; *t*_(18)_ = 3.03, *p* < 0.01; Supplementary Table 1). In addition, animal pictures elicited stronger activation than objects in the left anterior prefrontal cortex (PFC), left superior PFC, medial PFC, anterior cingulate cortex (ACC), angular gyrus and temporal gyrus (Figure 4). Note that animals and objects did not show a strong difference in cortical animate-inanimate networks (Martin, 2007) except that the right STS showed animals > objects (46, −56, 30; *t*_(18)_ = 2.49; *p* < 0.05, uncorrected), and the intraparietal lobe (IPL) showed objects > animals (24, −44, 44; *t*_(18)_ = 3.31; *p* < 0.005).

There was no significant main effect of context in the amygdala. The contextual effect was shown in the cortical regions such as the bilateral superior temporal gyrus/sulcus (STG/STS), cingulate gyrus and postcentral regions (Supplementary Table 1), which may be involved in evaluating biological threatening stimuli and relevance detection of social cues (Gross and Canteras, 2012; Adolphs, 2013; Rushworth et al., 2013).

### Interaction Between Category and Emotion

**Figure 5:**
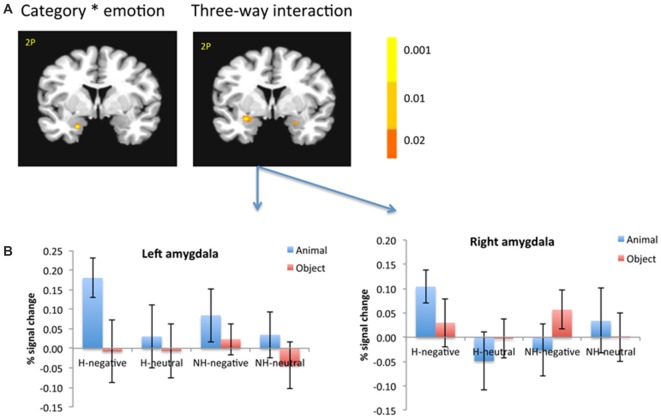
**The amygdala showed significant interaction between category and emotion, and three-way interaction (A).** The amygdala was selected from the significant three-way interaction, then the beta weights of each subject from the amygdala clusters were extracted and averaged across subjects **(B)**. The color bar represents *p*-values for *F*-statistics of interactions **(A)**. The left is on the left side. The error bars represent the standard errors of the means. The number/alphabet combinations at the top left corner of each brain image refer to the *y*-coordinates in the standard Talaraich atlas.

There was a significant interaction in the left amygdala between category and emotion (−19, −1, −21; *F*_(1,18)_ = 12.55; *p* < 0.005; Figure 5A, left). This interaction was shown as only negative pictures had stronger activation for animals than objects in the left amygdala (−26, 1, −9; *t*_(18)_ = 3.36; *p* < 0.005).

### Interaction Among Category, Emotion and Context

**Figure 6:**
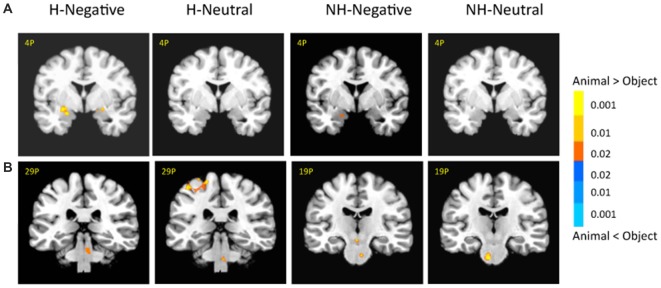
**There were different activation in the amygdala (A) and subcortical regions (B) under each condition.** The category effect was shown for negative pictures especially when the pictures had human contexts. The color bar represents *p*-values for *t*-statistics. The left is on the left side. The number/alphabet combinations at the top left corner of each brain image refer to the *y*-coordinates in the standard Talaraich atlas.

There was also a significant interaction of emotion, category and context in the bilateral amygdala (left: −26, −1, −11; *F*_(1,18)_ = 10.29; *p* < 0.01; right: 21, −4, −16; *F*_(1,18)_ = 6.62; *p* < 0.05; Figure 5A, right). The amygdala as regions of interest (ROIs) were selected from the significant three-way interaction. Then the beta weights of each subject from the amygdala clusters were extracted and averaged across the subjects. Figure 5B shows the signal changes for each category contrast from the amygdala ROIs. Consistently, the voxel-wise ANOVA showed that the category effect (animal > object) was significant for H-negative pictures bilaterally (left: 406 mm^3^, −26, −6, −6; *t*_(18)_ = 3.59; *p* < 0.005; right: 188 mm^3^, 24, −4, −9; *t*_(18)_ = 2.71; *p* < 0.02); and significant for NH-negative pictures in the left (126 mm^3^, −19, −4, −16; *t*_(18)_ = 2.72; *p* < 0.02; Figure 6A). No significant category effects for neutral conditions were found in the amygdala (*p’s* > 0.05).

In the brainstem, the category effect was significant in the anterior part for NH-pictures and in the posterior part for H-pictures (Figure 6B). In cortical regions, the left fusiform gyrus (−34, −42, −9; *t*_(18)_ = 4.99; *p* < 0.001) had stronger activation for H-negative animals (vs. objects). The left middle temporal gyrus (MTG; −39, −54, −7; *t*_(18)_ = 5.70; *p* < 0.001) and left PFC (−42, 21, 17; *t*_(18)_ = 5.06; *p* < 0.001) had stronger activation for NH-negative objects (vs. animals).

The three-way interaction was also manifested in the emotional effect, as the bilateral amygdala showed stronger activation for NH-negative objects vs. NH-neutral objects; (e.g., guns vs. hammer; left: −27, 1, −21; *t*_(18)_ = 3; *p* < 0.01; right: 28, 19, 1, −21; *t*_(18)_ = 3.55, *p* < 0.005), and stronger activation for H-negative animals vs. H-neutral animals (e.g., snake biting people vs. ladybug on the hand; left: −29, −4, −9; *t*_(18)_ = 2.99; *p* < 0.01; right: 21, −6, −9; *t*_(18)_ = 3.52; *p* < 0.005). When objects included human context or when animals did not include human context, negative and neutral pictures led to comparable activity in the amygdala.

The three-way interaction was also manifested in the context-related interaction, as the left amygdala showed stronger activation for H-negative animals than NH-negative ones (−22, −5, −12; *t*_(18)_ = 2.67, *p* < 0.02; e.g., snake biting people vs. snake alone).

### PPI Analysis for Emotional Effect and Category Effect

To determine the emotional network for unconscious processing, we performed a PPI analysis to identify regions showing activation correlated with amygdala activity. A right amygdala seed region was defined using the coordinates from the whole brain ANOVA main effect of emotion (21, −9, −9). The results showed that the activity of the right amygdala was positively correlated with that of the subcortical regions, including the caudate (−11, 11, −6; *t*_(18)_ = 4.68; *p* < 0.001) and thalamus (9, −21, 9; *t*_(18)_ = 3.75; *p* < 0.005). In addition, the right amygdala was positively correlated with the activity in cortical regions of medial PFC, ACC, PCC, supramarginal cortex, the fusiform gyrus (−47, −45, −24; *t*_(18)_ = 4.18; *p* < 0.001) and occipital regions (Supplementary Table 3). The results suggest that the subcortical network for processing negative pictures may be similar to that for negative faces (e.g., Morris et al., 1999; Williams et al., 2006).

To determine the category network (animal vs. object) for unconscious processing, we performed a second PPI analysis to identify regions showing activation correlated with amygdala activity. The right and left amygdala seed regions were defined using the coordinates from the ANOVA simple effect of category analysis. There were different correlation patterns between the amygdala and cortical regions for H-negative pictures and NH-negative pictures. The left amygdala showed positive correlation between amygdala and cortical regions of the middle PFC, ACC, PCC, inferior temporal gyrus and occipital regions for the NH-negative pictures (Table 2), suggesting that the amygdala has stronger correlation with these regions for animals (vs. objects). For the H-negative pictures, the left amygdala had positive correlation with the anterior PFC, superior PFC and middle temporal cortex for animals (vs. objects). In contrast to the result for NH-negative pictures, the left and right amygdala showed negative correlation with H-negative pictures in the thalamus, insula, medial prefrontal cortices and STG/STS (Table 3). This suggests that the amygdala has a stronger correlation with these regions for H-negative objects than for H-negative animals, leading to a decreased category effect when human contexts are involved.

**Table 2:** **PPI results for category effect (animal vs. object in NH-negative condition)**.

Animal vs. object (NH-negative)	L/R	Region	*t*-value	*x*	*y*	*z*
**Connectivity with left amygdala**
**Positive**
Frontal regions	R	ACC	4.61	14	34	−1
	L	Cingulate cortex	4.09	−11	9	29
	R	Middle frontal gyrus	4.13	34	36	−4
	L	Medial frontal gyrus	4.19	−6	21	46
	L	Medial frontal gyrus	4.29	−11	−6	51
	R	Medial frontal gyrus	4.41	11	41	16
	L	Medial frontal gyrus	4.05	−11	59	11
	R	Superior frontal gyrus	2.83	14	49	29
	L	Insula	5.24	−34	21	−1
	L	Insula	3.41	−34	−14	21
Parietal and midline	L	PCC/parietal cortex	4.63	−14	−51	16
	R	PCC	4.31	14	−49	41
	R	PCC	3.59	11	−51	6
	R	Paracentral gyrus	3.18	4	−31	44
Temporal regions	R	Inferior temporal gyrus	4.78	−56	−59	−6
	R	Inferior temporal gyrus	4.48	64	−21	−6
	R	STG	4.49	54	−24	6
	R	Fusiform gyrus	4.24	36	−57	−16
Occipital regions	L	Cuneus	3.54	−16	−76	6
	R	Lingual gyrus	4.00	21	−91	−1
	R	Lingual gyrus	3.31	1	−74	−4
Subcortical regions	L	Hippocampus	6.71	−26	−24	−4
**Negative** (not any)

**Table 3:** **PPI results for category effect (animal vs. object in H-negative condition)**.

Animal vs. object (H-negative)	L/R	Region	*t*-value	*x*	*y*	*z*
**Connectivity with left amygdala**
**Positive**
Frontal regions	R	Anterior prefrontal	4.40	49	29	−6
		cortex
	L	Superior frontal	3.15	−6	51	36
		cortex
Temporal regions	R	Inferior temporal	4.05	66	−11	−19
		gyrus
	L	Superior temporal	4.61	−54	−39	−1
		gyrus
**Negative**						
Frontal regions	R	Medial frontal	−5.87	11	−21	59
		gyrus
	L	Insula	−4.76	−31	−6	6
	L	Insula	−4.87	−39	1	16
	R	Insula	−4.10	41	16	16
Temporal regions	R	STG	−3.47	54	−41	14
Subcortical regions	L	Thalamus	−5.11	−6	−16	9
	R	Thalamus	−4.31	6	−19	9
**Connectivity with right amygdala**
**Positive** (not any)
**Negative**
Frontal regions	R	ACC	−5.21	6	14	31
	L	Cingulate	−4.33	−11	9	31
		cortex
	L	Inferior frontal	−3.21	−44	29	6
		gyrus
	L	Superior frontal	−3.54	−31	26	49
		gyrus
	L	Insula	−6.54	−31	−16	14
	L	Postcentral	−3.67	−51	−21	26
		cortex
	L	Precentral	−4.41	−34	−14	59
		cortex
	L	Precentral	−3.94	−44	−6	46
		cortex
Parietal regions	R	Supramarginal	−3.73	59	−46	34
		gyrus
	L	Inferior parietal	−3.36	−36	−44	54
		cortex
Temporal regions	R	STG	−5.16	59	−29	11
	L	STG	−4.81	−59	−24	11
	L	STG	−4.22	−54	−44	9
Occipital regions	R	Lingual gyrus	−3.87	24	−51	1
Subcortical regions	R	Thalamus	−4.41	6	−19	6
	L	Thalamus	−5.41	−11	−11	−9

## Discussion

The objective of this study was to explore whether different categories of emotional pictures elicited distinct brain activation without conscious awareness. By optimally controlling for factors of valence, arousal and familiarity of pictures across categories, we found significant interaction of context × category × emotion in the amygdala, showing that negative animal pictures elicited stronger activation in the amygdala than negative object pictures, especially for those with a human context. Animals and objects elicited limited activity in cortical animate-inanimate networks during unconscious processing. There were different correlation patterns between the amygdala and cortical regions for H-negative and NH-negative pictures. These results suggest that human context, emotional feature and stimulus category interact when pictures are processed without conscious awareness.

### Category Effect in the Amygdala and Subcortical Regions

The novel finding of our study was that the activity in the amygdala and subcortical regions showed significant three-way interaction among emotion, category and context. Specifically, enhanced activation for negative animals (vs. objects) occurred in the amygdala, especially for those with human context. This indicated that, when the stimuli are processed without conscious awareness, not only do negative pictures have an advantage in being processed, but also the negative animal pictures are more strongly processed, especially when the context contains human information.

The amygdala is part of an early vigilance system, detecting biologically relevant stimuli for further prioritized processing (Whalen, 1998; Davis and Whalen, 2001; Lipka et al., 2011). Under the assumption of the preparedness theory, negative stimuli in animal categories are processed unconsciously in the amygdala due to evolutionary pressure (Ohman and Mineka, 2001), but most studies only compared negative vs. neutral stimuli without taking the stimulus category as a factor. Our study provided direct neural evidence that, among the negative pictures, the animals was more processed in the amygdala than objects; probably due to the fact animals are potential predators for humans (Ohman and Mineka, 2001). Thus, the amygdala may help the perceptual system extract animal-related negative emotion information from an observed scene. It is of note that, only negative stimuli (but not neutral) showed significant activation in the amygdala, suggesting that the amygdala activation is not associated with general vigilance during unconscious processing, but rather preferentially involved in processing threatening conditions that are most relevant to human survival.

We also clarified that human context modulated the category effect in the amygdala. Previous studies have shown that human-related information may be unconsciously processed (Frith and Frith, 2008; Adolphs, 2010). Our study further demonstrated that, when human information was included in the context, the amygdala activation was stronger than when human context was not included, probably because of the more threatening nature of the human context. In contrast, the category effect in skin conductance appeared for pictures regardless of the human context involved (Tan et al., 2013). The difference between the fMRI activity and skin conductance results may have resulted from the fact that they reflect different aspects of emotional reactions. The amygdala activity reflects autonomic vigilance and preparation system especially for threatening animals with human contexts, and the SCR reflects action preparation for quick defensive response, which is not sensitive to contextual information without conscious awareness.

In addition to the amygdala, we found significant activation in subcortical regions, such as the PAG/LC and midbrain, in representing the category effect, which are known to be responsive not only to emotional faces (e.g., Williams et al., 2006), but also to other contents of emotional stimuli such as body gestures (de Gelder, 2006) and fearful animals (e.g., Carlsson et al., 2004; Alpers et al., 2009). As these stimuli belong to a category of biological stimuli, the amygdala coordinates with other subcortical regions in evaluating the biological significance of affective stimuli.

### Context-Modulated Connectivity Between the Amygdala and Cortical Regions

Context-dependent fear may have different neural mechanisms compared to context-independent fear, with more involvement or a different coupling pattern of the mPFC, ACC and other cortical regions (Gross and Canteras, 2012; Adolphs, 2013). The connectivity analysis elucidated that there were different correlation patterns for animal vs. object between the amygdala and cortical regions under the H-negative and NH-negative conditions. Positive correlation for NH-negative pictures revealed that the amygdala increased the response to animals relative to objects in these regions, leading to increased category effect (animal > object). The negative correlation for H-negative pictures, on the other hand, revealed that the amygdala connectivity with cortical and subcortical regions increased for objects (vs. animals), leading to reduced category effect. These regions included the aPFC, inferior PFC, ACC and insula, and they are related to responses to fear (e.g., increased attention for saliency; Williams et al., 2006; Carlson et al., 2012), affective autonomic feedback during emotional arousal (Carlson et al., 2012), modulatory control and regulation to emotional processing (Adolphs, 2013). This suggests that when negative objects with human contexts (e.g., gun held by a man) are presented, a top-down modulation from cortical regions would increase to prepare for quick responses. That is, the fear of evolutionary-related animals is automatic and prepared, whereas the fear of recently emerged objects may depend on their contexts (Ohman and Mineka, 2001; Blanchette, 2006; Yang et al., 2012a) and cortical interactions.

### Category Representation in Cortical Regions Without Conscious Awareness

In the first study that showed category effect for unconscious processing, we only found partial evidence for the category-specific activations. Among these previously revealed networks (Martin, 2007), we found that the right STS and left parietal cortex were differentially activated by the main effect of category. Semantic information is processed prior to emotion information during unconscious processing, because semantic categorization is faster and more accurate than affective processing even with short exposures (e.g., Nummenmaa et al., 2010). Nevertheless, semantic processing is not the same as processing under conscious conditions because stimuli are more coarsely and roughly processed. The subcortical pathway quickly processes coarse information of animate features, which may account for our behavioral findings, although detailed semantic information is not accessible for unconscious processing (Kang et al., 2011).

On the other hand, our study showed that the dorsal parts of the network, such as the STS and parietal cortex, were activated even without conscious awareness. It suggests that category representation in the dorsal pathway might not fully rely on conscious awareness. Previous studies have shown that the dorsal visual pathways may process information without conscious awareness (Goodale and Milner, 1992; Fang and He, 2005; Almeida et al., 2008; Suzuki et al., 2014; for review, Mahon and Caramazza, 2011; Milner, 2012). The suppressed stimuli may activate subcortical regions including the amygdala, and through direct projection from SC to posterior parietal cortex, activate dorsal regions (Almeida et al., 2008).

### Unconscious Processing of Category Information

The procedure and *post hoc* assessment ensured that participants processed the pictures unconsciously. For example, the sandwich-masking paradigm, in which the masks appeared before and after the target picture for an optimal masking effect, robustly reduced perceptual awareness (Harris et al., 2011). The results of the semantic tasks for assessing the level of awareness indicated that subjects processed the pictures without consciously identifying animals or objects.

The reasons that we chose the semantic judgment tasks were the following: (1) To achieve the main objective of the study, it was necessary to ensure that subjects processed the category/semantic information unconsciously. (2) The size judgment was also a type of semantic task because subjects had to make a decision based on the actual size of the stimulus (e.g., Dobbins et al., 2004; Kensinger and Schacter, 2008). Thus, even though they perceived a few perceptual features (e.g., color) and made inference in the animal/object judgment, they could not use the same information to make the size judgment (Tan et al., 2013). (3) Previous studies suggest that semantic judgment occurs earlier than emotional judgment due to the involvement of semantic categorization in affective analysis (Storbeck et al., 2006; Storbeck and Clore, 2007; Nummenmaa et al., 2010). If subjects were at chance to make categorization, it would be reasonable to assume that they were unaware of emotional attributes. Our previous behavioral studies showed that both semantic and emotional judgments were at chance when the same procedure was applied (Tan et al., 2013).

The category effect in our study may reflect the difference in semantic or conceptual representation between categories, rather than the differences in perceptual and other features for the following reasons. First, we obtained the category effect when the affective levels and familiarity levels were optimally matched across categories. There was no statistically significant difference between animals and objects in each condition (i.e., H-negative, NH-negative, H-neutral, NH-neutral) for both valence and arousal ratings. In addition, including more than one typical animal (e.g., snake, gun) enabled us to clarify to what extent animals differed from object in category level rather than individual concept level (e.g., snake). Second, low-level perceptual features, picture size, position of focal object and context and contextual information were matched across categories. In addition, previous studies suggest that category distinctions might be more dominant than perceptual features under unconscious conditions (e.g., Sebastiani et al., 2011; Codispoti et al., 2012). For instance, phobic patients only showed increased SCR for spiders (but not for crab and squirrel) when stimuli were unconsciously presented (Sebastiani et al., 2011). Third, affective analysis requires semantic categorization (Storbeck et al., 2006; Storbeck and Clore, 2007; Nummenmaa et al., 2010), which may precede affective evaluation of visual scenes. When the participants were presented with a scene for 40 ms, they extracted enough semantic information (Castelhano and Henderson, 2008). Consistently, the attention bias to the animal targets occured during the first fixation (Yang et al., 2012b). Combining with these findings, our results suggest that the amygdala and subcortical regions are important in detecting negative animal (but not objects) pictures so that humans are quickly prepared for potential dangers even without conscious awareness.

### Limitation

There are several potential limitations of the current study. First, emotional and contextual information should be rated to ensure that subjects were unaware of whether the picture was negative/neutral and whether it included human/human parts. Second, we did not included a “conscious” condition in the experimental design, which limits the possibility to draw any direct comparison between observed activation in this study with those that under a conscious condition. Third, our study showed that the amygdala was responsive to emotional, categorical and contextual features of pictures during unconscious processing. However, due to the temporal and spatial limitations of fMRI (Adolphs, 2010), we could not provide functional specialization with the amygdala from the current study. Fourth, although we explored category difference by controlling emotional and lower level differences, there are other ways to eliminate the potential confounding factors. For example, it is interesting to use identical pictures with only background replaced to explore the contextual effect, and to differentiate typical and atypical concepts to explore category effect under both conscious and unconscious conditions.

## Conclusion

When the valence and arousal levels were optimally controlled, the amygdala and subcortical regions showed stronger activation for animals than for objects, and this advantage was most prominent when the human context was involved. Furthermore, contextual information influenced their activities through top-down modulation, indicating that threatening stimuli that are most relevant to human survival are preferentially processed.

## Author Contributions

ZF: performed the experiments, analyzed the data, wrote the draft. HL: prepared the material, performed the experiments. GC: analyzed the data. JY: conceived and designed the experiments, analyzed the data, wrote the article.

## Funding

This research was supported by grants from the Global Research Initiative Program, National Institutes of Health (NIH), USA (R01TW007897, JY), and the National Science Foundation of China (31571114, JY).

## Conflict of Interest Statement

The authors declare that the research was conducted in the absence of any commercial or financial relationships that could be construed as a potential conflict of interest.
