# Supplementary material for: Unconscious Processing of Negative Animals and Objects: Role of the Amygdala Revealed by fMRI
Source: Front Hum Neurosci. 2016 Apr 5;10:146. doi: 10.3389/fnhum.2016.00146 (PMC4820445; doi:10.3389/fnhum.2016.00146)
Supplement: Supplementary file 1 [file Table_1.doc]

# STable 1 Main effect of emotion, category and context

| **Main effect of emotion** | L/R | Region | t-value | x | y | z |
| --- | --- | --- | --- | --- | --- | --- |
| **Negative > Neutral** |  |  |  |  |  |  |
| Frontal regions | L | Posterior cingulate cortex | 3.57 | -16 | -46 | 14 |
|  | R | Postcentral gyrus | 3.53 | 6 | -46 | 64 |
| Parietal regions | L | Precuneus | 4.68 | -11 | -44 | 61 |
| Temporal regions | R | Superior temporal cortex | 3.72 | 39 | -54 | 9 |
| Occipital regions | R | Cunes | 3.33 | 16 | -79 | 21 |
| Subcortical regions | L | Amygdala | 6.54 | -31 | -1 | -9 |
|  | R | Amygdala | 3.64 | 21 | -9 | -9 |
|  | R | Caudate | 4.03 | 14 | 16 | 9 |
|  | L | Thalamus | 3.53 | -14 | -9 | 1 |
|  | R | Superior Colliculus | 2.88 | 11 | -24 | -9 |
| **Negative < Neutral** |  |  |  |  |  |  |
| Occipital regions | R | Middle occipital gyrus | 3.35 | . | -81 | 11 |
|  |  |  |  |  |  |  |
| **Main effect of category** |  |  |  |  |  |  |
| **Animal > Object** |  |  |  |  |  |  |
| Frontal regions | L | Anterior prefrontal cortex | 5.17 | -49 | 39 | -1 |
|  | L | Superior frontal gyrus | 4.37 | -11 | 39 | 46 |
|  | L | Superior frontal gyrus | 4.05 | -31 | 16 | 54 |
|  | R | Superior frontal gyrus | 3.14 | 11 | 36 | 46 |
|  | L | Medial prefrontal cortex | 3.93 | -6 | 49 | 11 |
|  | R | Medial prefrontal cortex | 4.62 | 6 | -21 | 56 |
|  | R | Posterior cingulate cortex | 4.39 | 1 | -39 | 31 |
|  | L | Postcentral gyrus | 3.20 | -24 | -26 | 44 |
| Parietal regions | L | Angular gyrus | 5.70 | -46 | -64 | 34 |
| Temporal regions | R | Superior temporal gyrus | 2.49 | 46 | -56 | 30 |
|  | L | Inferior temporal gyrus | 4.01 | -51 | 1 | -16 |
|  | R | Inferior temporal gyrus | 4.10 | 61 | -6 | -16 |
|  | R | Anterior temporal gyrus | 6.18 | 51 | 14 | -21 |
| Subcortical regions | R | Substantia Nigra | 3.03 | 6 | -14 | -29 |
| **Animal < Object** |  |  |  |  |  |  |
| Frontal regions | R | Middle frontal gyrus | 4.31 | 29 | 39 | 19 |
|  | L | Inferior frontal gyrus | 4.05 | -24 | 39 | -1 |
|  | L | Insula | 3.27 | -34 | 21 | 14 |
| Parietal regions | L | Inferior parietal lobule | 3.31 | -24 | -44 | 44 |
| Occipital regions | L | Lateral occipital cortex | 4.12 | -29 | -76 | -4 |
|  |  |  |  |  |  |  |
| **Main effect of context** |  |  |  |  |  |  |
| **Human > Nonhuman** |  |  |  |  |  |  |
| Frontal regions | R | Cingulate cortex | 3.06 | 14 | -14 | 40 |
|  | L | Postcentral gyrus | 2.99 | -31 | -26 | 51 |
| Parietal regions | R | Precuneus | 3.83 | 21 | -46 | 46 |
| Temporal regions | L | mSTS/insula | 3.62 | -46 | -24 | 19 |
|  | R | Superior temporal gyrus | 3.42 | 46 | -29 | 11 |
| **Human < Nonhuman** |  |  |  |  |  |  |
| Occipital regions | L | Occipital gyrus | 3.11 | -49 | -54 | -16 |
|  | R | Occipital gyrus | 4.07 | 14 | -76 | -1 |
| Temporal regions | L | Fusiform gyrus | 4.57 | -39 | -44 | -14 |

# STable 2 Interaction among emotion, category and context

| **Context * Emotion** | L/R | | Region | F-value | x | y | z |
| --- | --- | --- | --- | --- | --- | --- | --- |
| Frontal regions | L | | ACC | 38.63 | -21 | 44 | 1 |
| Occipital regions | L | | Occipital gyrus | 15.28 | -4 | -71 | 1 |
|  |  | |  |  |  |  |  |
| **Context * Category** |  | |  |  |  |  |  |
| Frontal regions | L | | Middle frontal gyrus | 20.20 | -11 | -11 | 51 |
|  | L | | Insula | 20.37 | -36 | -16 | 26 |
|  | L | | Postcentral gyrus | 15.41 | -49 | -19 | 54 |
| Temporal regions | L | | Inferior temporal gyrus | 9.77 | -51 | -61 | -9 |
|  | L | | Fusiform gyrus | 12.66 | -31 | -66 | -14 |
|  | L | | Middel temporal gyrus | 13.64 | -36 | -59 | 4 |
| Occipital regions | R | | Occipitcal gyrus | 19.98 | 31 | -76 | 24 |
|  | L | | Occipitcal gyrus | 18.08 | -4 | -94 | -9 |
|  | R | | Occipitcal gyrus | 17.34 | 16 | -71 | 4 |
|  | R | | Occipitcal gyrus | 13.73 | 14 | -91 | 4 |
|  | L | | Occipitcal gyrus | 10.68 | -26 | -74 | 19 |
| Subcortical regions | L | | Caudate | 19.60 | -16 | 4 | 24 |
|  | R | | Caudate | 16.82 | 16 | -4 | 26 |
|  | L | | Midbrain | 12.89 | -4 | -31 | -29 |
|  | L | | Thalamus | 14.38 | -9 | -21 | 6 |
|  | R | | Thalamus/Pulvinar | 23.22 | 6 | -16 | 16 |
|  |  | |  |  |  |  |  |
| **Emotion * Category** | | |  |  |  |  |  |
| Temporal regions | L | | Fusiform gyrus | 19.75 | -31 | -61 | -11 |
|  | R | | Fusiform gyrus | 8.79 | 24 | -59 | -11 |
|  | R | | MTG | 21.64 | 56 | -49 | -9 |
|  | R | | STG | 16.19 | 61 | -49 | 16 |
| Occipital regions | L | | Occipital gyrus | 13.77 | 11 | -76 | -11 |
|  | R | | Occipital gyrus | 11.90 | 36 | -79 | 21 |
| Subcortical regions | L | | Amygdala | 10.69 | -19 | -11 | -4 |
|  | R | | Caudate | 17.46 | 14 | -4 | 19 |
|  | L | | Caudate | 12.22 | -19 | 16 | 4 |
|  | R | | PHG | 13.42 | 29 | -24 | -16 |
|  | L | | Putamen | 13.48 | -24 | 9 | 9 |
|  |  | |  |  |  |  |  |
| **Context * Emotion * Category** | | |  |  |  |  |  |
| Frontal regions | | L | Anterior frontal cortex | 24.45 | -49 | 39 | 9 |
|  | | R | Medial frontal cortex | 27.96 | 9 | -14 | 49 |
|  | | R | Inferior frontal gyrus | 14.31 | 49 | 19 | 39 |
|  | | L | Inferior frontal gyrus | 19.70 | -41 | 4 | 31 |
|  | | L | Inferior frontal gyrus | 10.24 | -49 | 16 | 24 |
| Temporal regions | | L | Fusiform gyrus | 24.98 | -44 | -44 | -9 |
|  | | R | STG | 14.47 | 59 | -41 | 19 |
|  | | L | STG | 28.47 | -61 | -44 | 21 |
| Parietal regions | | R | Intraparietal sulcus | 16.62 | 39 | -34 | 39 |
|  | | L | Intraparietal sulcus | 22.71 | -34 | -44 | 44 |
| Occipital regions | | L | Occipital gyrus | 13.78 | -29 | -89 | 6 |
| Subcortical regions | | L | Amygdala | 10.50 | -29 | 4 | -9 |
|  | | R | Amygdala | 18.13 | 24 | -4 | -4 |
|  | | R | Amygdala | 6.62 | 21 | -4 | -16 |
|  | | R | Midbrain | 15.76 | 29 | -61 | -24 |

# STable 3 PPI results for emotional effect (seed from Negative vs. neutral in left amygdala)

|  | L/R | Region | t-value | x | y | z |
| --- | --- | --- | --- | --- | --- | --- |
| **Positive** |  |  |  |  |  |  |
| Frontal regions | L | Middle prefrontal gyrus | 3.27 | -1 | -9 | 49 |
|  | L | Superior prefrontal gyrus | 3.97 | -14 | 61 | 16 |
|  | L | ACC | 4.04 | -9 | 24 | 34 |
|  | R | ACC/middle prefrontal gyrus | 5.65 | 9 | 29 | -1 |
| Parietal regions | R | PCC | 5.97 | 6 | -36 | 29 |
|  | R | Supramarginal gyrus | 6.26 | 41 | -46 | 29 |
| Temporal regions | L | STG | 4.22 | -54 | -59 | 29 |
|  | L | Fusiform gyrus | 4.14 | -47 | -45 | -24 |
| Occipital regions | R | Lingual gyrus | 4.28 | 21 | -84 | -1 |
|  | L | Lingual gyrus | 3.36 | -24 | -81 | 1 |
| Subcortical regions | L | Caudate | 4.68 | -11 | 11 | -6 |
|  | R | Thalamus | 3.75 | 9 | -21 | 9 |
| **Negative** |  |  |  |  |  |  |
| Frontal regions | L | Inferior frontal gyrus | 4.31 | 29 | 39 | 19 |
|  | L | Middle prefrontal gyrus | 4.05 | -24 | 39 | -1 |
|  | L | Superior prefrontal gyrus | 3.27 | -34 | 21 | 14 |
